# Novel knowledge-based mean force potential at the profile level

**DOI:** 10.1186/1471-2105-7-324

**Published:** 2006-06-27

**Authors:** Qiwen Dong, Xiaolong Wang, Lei Lin

**Affiliations:** 1 School of Computer Science and Technology, Harbin Institute of Technology, Harbin, PR China

## Abstract

**Background:**

The development and testing of functions for the modeling of protein energetics is an important part of current research aimed at understanding protein structure and function. Knowledge-based mean force potentials are derived from statistical analyses of interacting groups in experimentally determined protein structures. Current knowledge-based mean force potentials are developed at the atom or amino acid level. The evolutionary information contained in the profiles is not investigated. Based on these observations, a class of novel knowledge-based mean force potentials at the profile level has been presented, which uses the evolutionary information of profiles for developing more powerful statistical potentials.

**Results:**

The frequency profiles are directly calculated from the multiple sequence alignments outputted by PSI-BLAST and converted into binary profiles with a probability threshold. As a result, the protein sequences are represented as sequences of binary profiles rather than sequences of amino acids. Similar to the knowledge-based potentials at the residue level, a class of novel potentials at the profile level is introduced. We develop four types of profile-level statistical potentials including distance-dependent, contact, Φ/Ψ dihedral angle and accessible surface statistical potentials. These potentials are first evaluated by the fold assessment between the correct and incorrect models generated by comparative modeling from our own and other groups. They are then used to recognize the native structures from well-constructed decoy sets. Experimental results show that all the knowledge-base mean force potentials at the profile level outperform those at the residue level. Significant improvements are obtained for the distance-dependent and accessible surface potentials (5–6%). The contact and Φ/Ψ dihedral angle potential only get a slight improvement (1–2%). Decoy set evaluation results show that the distance-dependent profile-level potentials even outperform other atom-level potentials. We also demonstrate that profile-level statistical potentials can improve the performance of threading.

**Conclusion:**

The knowledge-base mean force potentials at the profile level can provide better discriminatory ability than those at the residue level, so they will be useful for protein structure prediction and model refinement.

## Background

The development and evaluation of new energy functions is critical to the accurate modeling of the properties of biological macromolecules [1]. A potential that can discriminate between the native and miss-folded structures is crucial for any protein structure prediction protocol to be fully successful. Toward this end, two different types of potential functions are currently in use [2-4]. The first class of potentials, the so-called physical-based potential, is based on the fundamental analysis of forces between atoms [5-7]. The second class, the so-called knowledge-based potentials, extracts parameters from experimentally solved protein structures [8-11]. The advantage of the first class of potentials is that, in principle, they can be derived from the laws of physics. The disadvantage is that the calculation of free energy is very difficult because the computation should include an atomic description of the protein and the surrounding solvent. Currently this type of computation is generally too expensive for protein folding [12]. While, with today's computer resources, knowledge-based potentials can be quite successful at fold recognition [13] and ab initio structure prediction [14,15].

Much can be learned through statistical analysis of interacting groups in experimentally determined protein structures. Such analysis provides the basis for knowledge-based potentials of mean force. Generally, knowledge-based potentials have used a simple one- or two-point-per-residue representation, which results in the potentials at the residue level. Each residue in a protein sequence is represented by one or two points in three-dimensional space. These points are usually located at the coordinates of each residue's C_α _atoms, C_β _atoms or at the coordinates of the center of each side chain. Discrimination is based on each residue's preference to be buried or exposed [16], its preference for a particular secondary structure conformation [17], its preference for the contact number with other residues [18] and its preference to be in contact at a particular distance and sequence separation from other residues [19,20]. However, to capture the finer details of atom-atom interactions in proteins, a more detailed description is necessary. Each heavy atom either at the main-chain or side-chain is represented by an independent point, which results in the knowledge-based potentials at the atom level. A number of potentials at the atom level have been designed [21-24]. Because of its atom level definition, the knowledge-based potentials at the atom level can provide better discriminatory power than obtained at the residue level [25].

Although the knowledge-based mean force potentials at the residue level are based on the coarse description of protein structures, they are easier to be used in fold recognition or threading than those at the atom level [22]. Many fold-recognition methods use knowledge-based potentials to interpret probabilistic scoring functions. Sequence-template alignments are evaluated in terms of a scoring function and the score of the alignment is interpreted as a "free energy" of the sequence in the conformation imposed by the alignment [26]. This interpretation indicates that the most probable sequence-structure alignment is the one with the lowest "free energy". The 123D method [27] applies the pairwise sequence alignment and contact capacity potentials to fast protein fold recognition. The SPARK method [18] combines the sequence-profile alignment and single-body knowledge-based energy score for fold recognition. The GenTHREADER method [28] apply neural network to evaluate the compatibility of the sequence and the template with pairwise potentials and solvation potentials as input. In addition to the fold recognition or threading, knowledge-based potentials are widely used in selection of native structures of proteins [29,30], estimation of protein stability [31], ab initio protein structure prediction [32-34], etc.

The aim of this paper is to develop a class of novel knowledge-based mean force potentials at the profile level, which uses the evolutionary information of the profile [35]. Such potentials can provide better discriminatory power than those at the residue level and can be incorporated into the process of fold recognition or threading. Multiple sequences alignments of protein sequences may contain much information regarding evolutionary processes. This information can be detected by analyzing the output of PSI-BLAST [35,36]. The frequency profiles are directly calculated from the multiple sequence alignments and then converted into binary profiles with a cut-off probability for usage. Such binary profiles make up of a new alphabet for protein sequences. Similar to the knowledge-based potentials at the residue level, a class of novel potentials at the profile level is introduced. We developed four types of profile-level statistical potentials including distance-dependent, contact, Φ/Ψ dihedral angle and accessible surface statistical potentials. These potentials are first evaluated by the fold assessment between the correct and incorrect models generated by comparative modeling. They are then used to recognize the native structure from the well-constructed decoy set. Experimental results show that all the knowledge-base mean force potentials at the profile level outperform those at the residue level.

## Results

### Fold assessment on test models

To evaluate the performance of the statistical potentials at the profile level and those at the residue level, the first experiment is made to discriminate between the good models and the bad models on our structure models. The parameters of various potentials are selected as the optimal values as suggested by others [11,18]. For the distance-dependent potentials, the interaction center is set as C_β _atom. The distance range is 30 Å with distance interval of 1 Å. The sequence separation *k *varies from 3 to 9. The rare situation with sequence separation larger than 9 is included in the last bin. For the contact potential, the number of contact bin is set to 25. In the rare occasions of more than 25 contacts, the statistics are included in the bin for 25 contacts. All the contacts with sequence separation larger than 1 are computed. For the Φ/Ψ dihedral angle potential, each of the torsion is divided into 36 bins. There are total 1296 bins. For the accessible surface potential, the interaction center is set as C_β _atom. The distance range (the radius of the sphere) is set as 9 Å. The burial range varies from 0 to 40 atoms with burial interval of 2 atoms. The atoms within the same residues are not considered for statistics.

The statistics of propensity of various potentials are performed on the PDB25 dataset. These potentials are then calculated to discriminate between good models and bad models for each of the sequence. The fraction of correctly predicted case (CP), the success rates, the Z-scores and the ROC curve are employed to evaluate the performance. The results are shown in table 1 and Fig. 2. In the ROC curve, a lower plot corresponds to a better discriminative power.

As can be seen, all the knowledge-based mean force potentials at the profile level outperform those at the residue level. The improvements of various potentials at the profile level are different from those at the residue level. Significant improvements of CP are obtained for the distance-dependent and accessible surface potentials (5–6%). The contact and Φ/Ψ dihedral angle potential only get a slight improvement of CP (1–2%).

### Tested on Baker's set

The Baker's set [37] is a well-constructed decoy set that is obtained by large-scale comparative modeling. The dataset consists of 41 single domain proteins and each protein is attached with about 1400 decoy structures. The decoy structures are classified into good models and bad models by the same criterion as used by our structure models. Models with >30% structural overlap with the experimentally determined structures are grouped into good models. Models with <15% structural overlap with the experimentally determined structures are grouped into bad models. The fold assessment results are shown in table 2.

Overall the knowledge-based mean force potentials at the profile level still outperform those at the residue level. Significant improvements are obtained for the distance-dependent and accessible surface potentials. The CP scores of all potentials on the Baker's set are lower than those on our structure models. There are two reasons for this phenomenon. The first one is that the Baker's set is inherently difficult to discriminate. Such dataset is carefully constructed and satisfies the so-called four criteria listed in their introduction [37]. The second one is that the number and distribution of good models and bad models in this dataset are different from those in our dataset. In Baker's dataset, the total models for a sequence are very large (more than one thousand) and the distribution between the number of good models and that of bad models is different. For example, the sequence 1ptq has only 8 good models and 1647 bad models, while the sequence 1res has 1722 good models and only one bad model. In our dataset, each sequence has about thirty models and the good models and bad models are equally distributed (about fifteen respectively).

### PROSTAR decoy set evaluation

All the decoy sets from PROSTAR website [38] are well-constructed and widely used for evaluation of all kinds of newly developed potentials [21,24]. Three subsets including MISFOLD [39], IFU [40] and PDBERR [38] are selected for testing. The IFU dataset contains a set of models for small peptides rather than the whole protein chains. Since direct generation of profiles for such small peptides may not be reliable, we first generate the profiles of the whole protein chains and extract the corresponding profiles for such small peptides. There are two proteins (3SNS, 1ILB) that are not found in the PDB database [41], the corresponding decoy models are removed (3SNS_16-29, 3SNS_6-21, 1ILB_99-110). The results of decoy set evaluation are given in table 3. When the energy Z-scores of the native structure are lower than those of the decoy models, a correct discrimination is obtained.

All the knowledge-based mean force potentials get good results on the MISFOLD and PDBERR dataset and acceptable results on the IFU dataset. The IFU dataset is more challenging than the other two dataset, because this dataset contains the decoy models for small peptides and fold assessment by statistical potentials is most difficult for the very small models [11]. Small models are difficult to assess because of the relatively small number of pairwise interactions by which they are judged, not because of their incompleteness. Overall, the potentials at the profile level still outperform those at the residue level on the IFU dataset. The best discrimination is achieved by the distance-dependent potentials at the profile level, which correctly recognize 35 out of 41 decoy pairs, corresponding to accuracy of 85%. Such results outperform other atom-level potentials such as the Residue specific all-Atom Probability Discriminatory Function (RAPDF) [21] and the atomically detailed potentials of T32S3 [24]. These two potentials get 100% accuracy on the MISFOLD dataset as done by the profile-level distance-dependent potentials. They correctly identified 73% and 80% of the decoy pair on the IFU dataset respectively [24], while the profile-level distance-dependent potentials correctly identified 85% of the decoy pair on the same dataset.

### Multiple decoy sets evaluation

To give an un-bias result and fair comparison with other potentials, we use five out of seven multiple decoy sets as used by Zhang et al. [42]. They include the 4state_reduce set [43], lmds set [44], fisa set [14], fisa_casp3 set [45], lattice_ssfit set [46]. Totally, there are 32 multiple decoy sets available (listed at Table 1 of Zhang et al. [42]). No decoy structures in the original decoy sets are omitted in this study. The diverse and comprehensive decoy sets ensure the fair evaluation of the overall quality of the potentials. We also compare our potentials with DFIRE-SCM [42], which is one of the most recent residue-level potentials. The results are evaluated in terms of success rates in native discriminations and Z-score for different decoy sets. The performances of different potentials are shown in Table 4.

As can be seen, all the profile-level statistical potentials outperform those at the residue-level. Overall, the success rates of profile-level potentials are better than those of residue-level potentials. Even with the same success rates on some datasets, the Z-scores of profile-level potentials are higher than those of residue-level. The distance-dependent knowledge-based potential [19] in this paper is the ProsaII potential as mentioned by Zhang et al. [42], which is inferior to DFIRE-SCM according to Zhang et al. [42]. The distance-dependent potential at the profile level is comparable with the DFIRE-SCM potential. The former correctly recognizes 22 out of 32 decoy structures, while the latter correctly recognizes 23 out of 32 decoy structures. The contact, Φ/Ψ dihedral angle and accessible surface statistical potentials are single-body residue-level statistical potentials, which are based on the coarse descriptions of protein structures. Such potentials get lower performance in comparison with other two-body atom-level statistical potentials in many experiments [21,25]. These simple potentials at the profile-level still outperform those at the residue-level according to our experiments. These results suggest that the binary profiles are smarter representations of protein structures than residues.

## Discussion

### The probability threshold has not significant influence on the profile-level statistical potentials

The frequency profiles are calculated from the multiple sequence alignments outputted by PSI-BLAST [35] and converted into binary profiles by a probability threshold *P*_*h*_. The total number of binary profiles is dependent on the size of the database and the value of probability threshold *P*_*h*_. Since each combination of the twenty amino acids corresponds to a binary profile and vice versa, the total number of binary profiles is 2^20. In fact, only a small fraction of binary profiles appear. These binary profiles substitute for novel alphabets of protein sequences to develop a class of novel profile-level statistical potentials. Since the probability threshold *P*_*h *_is a parameter, it needs to be optimized. The results are shown in table 5. We surprisingly found that the probability threshold *P*_*h *_has not significant influence on all the profile-level statistical potentials. When the probability threshold is larger than 0.28, the number of binary profiles is very small and the discriminative power of all the profile-level statistical potentials drops quickly. Since the decrease in the number of residue types reduces the discriminative ability of the potentials [11], we can draw a similar conclusion that an increase in the number of alphabets of protein sequences can improve the discriminative power of the potentials. This study provides a method for increasing the number of alphabets of protein sequences, that is, the profile method.

### The energy of profile-level statistical potentials correlates well with RMSD

Another measure of the potential quality and its global attraction is the dependence of the energy on the proximity to the native structure. The proper coordinate to measure proximity to the native structure is not obvious. However in numerous cases the RMSD is used [47]. In Fig. 3, the scatter plot of the energy as a function of the decoy Cα RMSD value is plotted. Since the potentials of different native structures are not comparable, only one of the sequence (1vcc) and its models are plotted. As can be seen, the energy of profile-level statistical potentials correlates well with the Cα RMSD up to quite large RMSDs. This suggests that the profile-level potentials can be useful in simulations that attempt to get closer to the native conformation starting from a distant conformation.

### Using evolutionary information can improve the discriminative power of knowledge-based mean force potentials

In the profile-level statistical potentials, the protein sequence is represented as a sequence of frequency profile rather than an amino acid sequence. The frequency profile contains the evolutionary information of protein sequences, which is the probabilities of the amino acids occurred in the specific position of the protein sequences. Such profiles are used to produce more discriminative potentials. As the best of our knowledge, this is the first usage of evolutionary information for developing more advanced potentials. The potentials at the profile level are prior to those at residue level according to the experiments. So evolutionary information can improve the discriminative power of knowledge-based mean force potentials. This conclusion is not surprising, since the evolutionary information is widely used in lots of biological problems such as the protein secondary structure prediction [48,49], remote homologue detection [50,51], sub-cellular localization [52,53], domain boundary prediction [54], fold recognition [55], protein-protein interaction prediction [56], function annotation [57], etc.

### Profile-level statistical potentials can improve the performance of threading

Fold recognition or threading is another application of knowledge-based mean force potentials. Many methods combine the residue-level statistical potentials with sequence alignments for threading, such as the SPARKS method [18]. We have implemented a threading method that combines the profile-level statistical potentials with profile-profile alignments. Such profile-level threading method (referred as profile-threading) is compared with the threading method that uses the residue-level statistical potentials (referred as residue-threading).

Since the multi-body statistical potentials are hard to be used for threading, a combined potentials has been presented, which integrate the three single-body potentials of this study, that is, the Φ/Ψ dihedral angle, accessible surface and contact statistical potentials:

*E*(*i*) = *E*^*t*^(*i*, *φ*_*i*_, *ϕ*_*i*_) + *w*^*f *^*E*^*f*^(*i*,*S*_*i*_) + *w*^*c *^*E*^*c*^(*i*,*N*_*i*_)     (14)

where *E*^*t*^, *E*^*f*^, E^*c *^is the Φ/Ψ dihedral angle, accessible surface and contact statistical potentials respectively, *i *is amino acid for residue-level potentials and profile for profile-level potentials at the i-th position of the sequence, *w*^*f *^and *w*^*c *^are the weights of accessible surface and contact statistical potentials. The total potential for a protein is then obtained by summing the potentials of each of the amino acid or profile. Using the decoy set of PROSTAR, the optimal parameters of *w*^*f *^and *w*^*c *^for residue-level potential are selected as 0.5 and 3.375, which correctly identifies 59 out of 69 decoy pairs. The optimal parameters of *w*^*f *^and *w*^*c *^for profile-level potential are selected as 1 and 2.5, leading to correctly identify 62 out of 69 decoy pairs.

The profile-profile alignment method used here is the PICASSO3 method [58], which gives the best results of fold recognition [59]. The profile-profile score to align the position *i *of a sequence *q *and the position *j *of a template *t *is given by:

mij=−∑k=120[fikqSjkt+Sikqfjkt]     (15)
 MathType@MTEF@5@5@+=feaafiart1ev1aaatCvAUfKttLearuWrP9MDH5MBPbIqV92AaeXatLxBI9gBaebbnrfifHhDYfgasaacH8akY=wiFfYdH8Gipec8Eeeu0xXdbba9frFj0=OqFfea0dXdd9vqai=hGuQ8kuc9pgc9s8qqaq=dirpe0xb9q8qiLsFr0=vr0=vr0dc8meaabaqaciaacaGaaeqabaqabeGadaaakeaacqWGTbqBdaWgaaWcbaGaemyAaKMaemOAaOgabeaakiabg2da9iabgkHiTmaaqahabaWaamWaaeaacqWGMbGzdaqhaaWcbaGaemyAaKMaem4AaSgabaGaemyCaehaaOGaem4uam1aa0baaSqaaiabdQgaQjabdUgaRbqaaiabdsha0baakiabgUcaRiabdofatnaaDaaaleaacqWGPbqAcqWGRbWAaeaacqWGXbqCaaGccqWGMbGzdaqhaaWcbaGaemOAaOMaem4AaSgabaGaemiDaqhaaaGccaGLBbGaayzxaaaaleaacqWGRbWAcqGH9aqpcqaIXaqmaeaacqaIYaGmcqaIWaama0GaeyyeIuoakiaaxMaacaWLjaWaaeWaaeaacqaIXaqmcqaI1aqnaiaawIcacaGLPaaaaaa@5882@

where *f*_*ik*_^*q*^, *f*_*ik*_^*t*^, *S*_*ik*_^*q *^and *S*_*ik*_^*t *^are the frequencies and the position-specific score matrix (PSSM) scores of amino acid *k *at position *i *of a sequence *q *and position *j *of a template *t*, respectively.

The profile-profile alignment is combined with the knowledge-based score for threading. The total score is given by:

*u*^*total *^= *m*_*ij *_+ *w*^*s *^*E*_*j *_(*s*_*i*_)

where *E*_*j*_*(s*_*i*_*) *is the combined potentials score of the template at position *j *with the residue type (for residue-threading) or profile type (for profile-threading) *s*_*i *_of the position *i *of the query sequence, *w*_*s *_is the weight factors for structure scores. The dynamic programming algorithm is employed to find the minimum of the total score of the sequence-template alignments.

The HOMSTRAD database [60] is selected to test the alignment accuracy of the two threading methods. Only families containing two single-chain sequences and with sequence identities less than 40% are considered. The resulting dataset contains 390 families and is randomly divided into training set and test set with ratio of 4:1. The genetic algorithm is used to find the optimal parameters on the training set including the structure factor *w*_*s*_, the gap-open penalty *w*_*0 *_and the gap-extension penalty *w*_*1*_. Such parameters are then applied to test the alignment accuracy on the test set. The results are shown in Table 6. The profile-level threading method outperforms the residue-level threading method, so profile-level statistical potentials can improve the performance of threading.

## Conclusion

In this study, a class of novel knowledge-based mean force potentials at the profile level has been presented. The frequency profiles are directly calculated from the multiple sequence alignments outputted by PSI-BLAST and converted into binary profiles with a probability threshold. Such binary profiles make up of a new alphabet for protein sequence. Because the binary profiles contain evolutionary information, they provide better descriptions of protein structures than the residues. We develop a class of novel statistical potentials at the profile level. Fold assessment and decoy sets evaluation results show that the statistical potentials at the profile-level outperform those at the residue level. Future work will aim at application of the profile-level statistical potentials to protein structure prediction and exploring other applications of such binary profiles such as remote homology detection, prediction of protein class etc.

## Methods

### Dataset

To evaluate the usefulness of the statistical potentials, large sets of protein structure models are needed [61]. Three datasets are used in this study. The first one is our structure models generated by large-scale comparative modeling [62]. The second one is the Baker's models [37] that also produced by comparative modeling. The third one is the PROSTAR decoy set [38]. The three datasets are briefly described as follows.

The freely available software MODELLER [62] is used for comparative modeling. The protein chains of the Protein Data Bank (PDB) [41] are downloaded from the SCOP database [63]. The sequence set and the template set are taken from the ASTRAL compendium [64] with sequence identity less than 40% and 80% respectively. Two sets of models including good models and bad models are calculated by large-scale comparative modeling. The models are classified depending on their structural similarity to the actual structure of the target protein. The good models are built on the basis of the correct templates and the structure-structure alignments between the target sequences and the template structures. The correct templates mean that the target sequences and the template structures share the same fold. The structure-structure alignment method is the iterative least-squares superposition method implemented by the MODELLER package [62]. Models with <30% structural overlap with the actual experimentally determined structure are eliminated. Structural overlap [11] is defined as the fraction of the equivalent C_*α *_atoms upon least-squares superposition of the two structures with the 3.5 Å cutoff. The final set contains 4207 good models. The bad models are built on the basis of templates with incorrect folds but correct alignments (the structure-structure alignment) or the templates with correct folds but incorrect alignments (the sequence-sequence alignments). Models with >15% structure overlap with the actual target structure are eliminated. The final set contains 7045 bad models.

The Baker set [37] currently consists of 41 single domain proteins with varying degrees of secondary structures and lengths from 25 to 87 residues. Each protein is attached with about 1400 decoy structures generated by ab initio protein structure prediction method of Rosetta [45]. This set provides a good challenge for scoring functions and selection schemes to test themselves against the local minima around the native state. The Baker set can be downloaded from  using the link "Download the all atom decoys used by Tasi et al. (pdbs)".

The PROSTAR set contains a set of well-constructed decoy sets. Three subsets including MISFOLD, IFU and PDBERR are selected for testing the performance of potentials. Each decoy set contains one correct and one or more incorrect or approximate conformations. The MISFOLD decoy set [39] consists of 25 examples of pairs of proteins with the same number of residues in the chain, but different sequences and conformations. The IFU decoy set is based on a set of 44 peptides that are proposed to be independent folding units as determined by local hydrophobic burial and experimental evidence [40]. The PDBERR decoy set is comprised of three structures determined using X-ray crystallography which are later found to contains errors and the corresponding correct experimental conformations [38].

### Known structures for calculating potentials

The database of proteins used for the statistical analysis of various potentials is a subset of PDB database [41] obtained from the PISCES [65] web-server. The representative structures are selected such that they share <25% sequence identity with each other and better than 2.5 Å resolutions. The structures that contain missing atoms and chain breaks are excluded. We also remove the overlapped protein chains that are used in the three decoy sets. The resulting database contains 2352 chains and refers to PDB25 dataset.

### Generating and converting of profiles

The PSI-BLAST [35] is used to generate the profiles of amino acid sequences with the default parameter values except that the number of iterations is set to 10. The search is performed against the NR90 database that is obtained by culling the NR database of NCBI using the Perl script from EBI [66]. The redundant sequences with sequence identity larger than 90% are removed. The frequency profiles are directly obtained from the multiple sequence alignments outputted by PSI-BLAST. The target frequency reflects the probability of an amino acid occurrence in a given position of the sequences. The method of target frequency calculation is similar to that implemented in PSI-BLAST. The multiple sequence alignments are used to calculate the frequency profiles. The sequence weight is assigned by the position-based sequence weight method [67]. Since calculation of target frequencies from the multiple sequence alignments may be influenced by a lot of factors including small sample size [68] and prior knowledge of relation among the residues [69,70]. We have implemented the data-dependent pseudo-count method to estimate the target frequencies [69]. Given the observed frequency of amino acid *i *(*f*_*i*_) and the background frequency of amino acid *i *(*p*_*i*_), the pseudo-count for amino acid *i *is computed as follows:

gi=∑j=120fj*(qij/pj)     (1)
 MathType@MTEF@5@5@+=feaafiart1ev1aaatCvAUfKttLearuWrP9MDH5MBPbIqV92AaeXatLxBI9gBaebbnrfifHhDYfgasaacH8akY=wiFfYdH8Gipec8Eeeu0xXdbba9frFj0=OqFfea0dXdd9vqai=hGuQ8kuc9pgc9s8qqaq=dirpe0xb9q8qiLsFr0=vr0=vr0dc8meaabaqaciaacaGaaeqabaqabeGadaaakeaacqWGNbWzdaWgaaWcbaGaemyAaKgabeaakiabg2da9maaqahabaGaemOzay2aaSbaaSqaaiabdQgaQbqabaaabaGaemOAaOMaeyypa0JaeGymaedabaGaeGOmaiJaeGimaadaniabggHiLdGccqGGQaGkcqGGOaakcqWGXbqCdaWgaaWcbaGaemyAaKMaemOAaOgabeaakiabc+caViabdchaWnaaBaaaleaacqWGQbGAaeqaaOGaeiykaKIaaCzcaiaaxMaadaqadaqaaiabigdaXaGaayjkaiaawMcaaaaa@4972@

where *q*_*ij *_is the score of amino acid *i *being aligned to amino acid *j *in BLOSUM62 substitution matrix that is the default score matrix of PSI-BLAST.

The target frequency is then calculated as:

*Q*_*i *_= (*αf*_*i *_+ *βg*_*i*_)/*α *+ *β *    (2)

where *α *is the number of different amino acids in a given column minus one and *β *is a free parameter set to a constant value of 10, the value initially used by PSI-BLAST.

**Figure 1:**
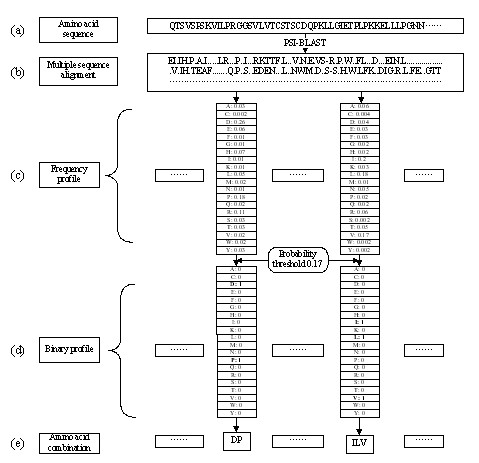
**The process of calculating frequency profiles and converting it into binary profiles**. (a) For a given amino acid sequence, (b) the multiple sequence alignment is obtained by PSI-BLAST. (c) The frequency profile is calculated on the multiple sequence alignment and (d) transforms into a binary profile with a probability threshold. (e) A substring of amino acid combination is then obtained by collecting the binary profile with non-zero value for each position of the protein sequences.

**Figure 2:**
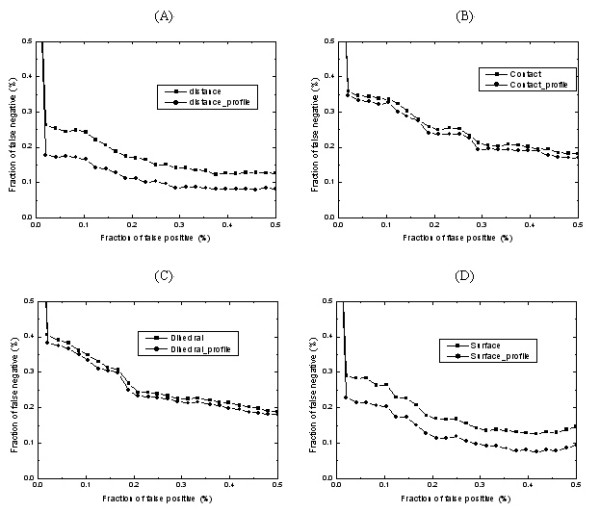
**ROC curves of various potentials tested on our structure models**. The lower the curve, the better the discrimination between the good and bad models. Subfigure (A), (B), (C) and (D) show the performance of residue-level and profile-level potentials of distance-dependent, contact, Φ/Ψ dihedral angle and accessible surface statistical potentials respectively. The potentials with _profile suffix indicate the corresponding potentials at the profile level.

**Figure 3:**
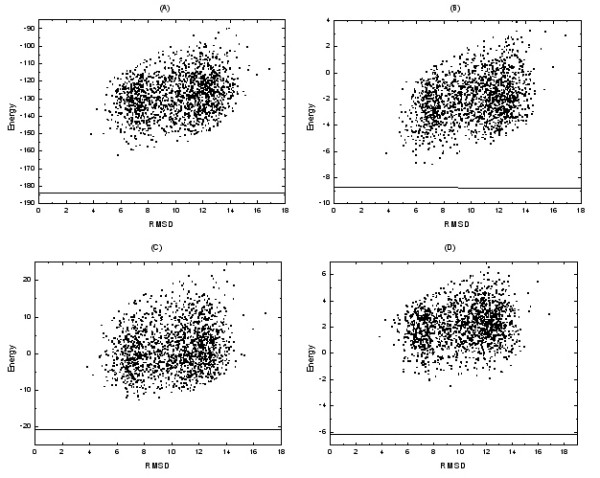
**A scatter plot of energy versus RMSD**. A horizontal line highlights the score of the native state. Subfigure (A), (B), (C) and (D) show the correlation of profile-level potentials of distance-dependent, contact, Φ/Ψ dihedral angle and accessible surface statistical potentials respectively. The total number of structure models included in each plot is 1858. Shown in the plot are the structure models of the sequence 1vcc from Baker's dataset.

Because the frequency profile is a matrix of frequencies for all amino acids, it cannot be used directly and need to be converted into a binary profile by a probability threshold *P*_*h*_. When the frequency of an amino acid is larger than *P*_*h*_, it is converted into an integral value of 1, which means that the specific amino acid can occur in a given position of the protein sequences during evolution. Otherwise it is converted into 0. A substring of amino acid combination is then obtained by collecting the binary profile with non-zero value for each position of the protein sequences. These substrings have approximately represented the amino acids that possibly occur at a given sequence position during evolution. Each combination of the twenty amino acids corresponds to a binary profile and vice versa. Fig. 1 has shown the process of generating and converting the profiles.

### Knowledge-based mean force potentials

Similar to the knowledge-based potentials at the residue level, a class of novel potentials at the profile level is introduced. We developed four types of profile-level statistical potentials including distance-dependent, contact, Φ/Ψ dihedral angle and accessible surface statistical potentials. The difference between the potentials at the residue level and those at the profile level is that each residue is represented as a binary profile rather than a single residue. For the residue-level statistical potentials, the interaction types are the 20 standard amino acids. While for the profile-level statistical potentials, the interaction types are the binary profiles. Other parameters are same for the two kinds of statistical potentials. Such representation contains evolutionary information and provides more discriminative power than the single residue according to the experimental results.

#### Distance-dependent potential

The distance-dependent statistical potentials are calculated as described in [20,22]. The energy of two interaction types (*ij*) with sequence separation *k *and distance interval *l *is given by:

Ekij(l)=RTln⁡[1+Mijkσ]−RTln⁡[1+Mijkσfkij(l)fkxx(l)]     (3)
 MathType@MTEF@5@5@+=feaafiart1ev1aaatCvAUfKttLearuWrP9MDH5MBPbIqV92AaeXatLxBI9gBaebbnrfifHhDYfgasaacH8akY=wiFfYdH8Gipec8Eeeu0xXdbba9frFj0=OqFfea0dXdd9vqai=hGuQ8kuc9pgc9s8qqaq=dirpe0xb9q8qiLsFr0=vr0=vr0dc8meaabaqaciaacaGaaeqabaqabeGadaaakeaacqWGfbqrdaqhaaWcbaGaem4AaSgabaGaemyAaKMaemOAaOgaaOGaeiikaGIaemiBaWMaeiykaKIaeyypa0JaemOuaiLaemivaqLagiiBaWMaeiOBa4Maei4waSLaeGymaeJaey4kaSIaemyta00aaSbaaSqaaiabdMgaPjabdQgaQjabdUgaRbqabaacciGccqWFdpWCcqGGDbqxcqGHsislcqWGsbGucqWGubavcyGGSbaBcqGGUbGBcqGGBbWwcqaIXaqmcqGHRaWkcqWGnbqtdaWgaaWcbaGaemyAaKMaemOAaOMaem4AaSgabeaakiab=n8aZnaalaaabaGaemOzay2aa0baaSqaaiabdUgaRbqaaiabdMgaPjabdQgaQbaakiabcIcaOiabdYgaSjabcMcaPaqaaiabdAgaMnaaDaaaleaacqWGRbWAaeaacqWG4baEcqWG4baEaaGccqGGOaakcqWGSbaBcqGGPaqkaaGaeiyxa0LaaCzcaiaaxMaadaqadaqaaiabiodaZaGaayjkaiaawMcaaaaa@6DDE@

where *M*_*ijk *_is the number of occurrences for the interaction type pair *ij *separated by *k *residues in sequence:

Mijk=∑l=1nf(i,j,k,l)     (4)
 MathType@MTEF@5@5@+=feaafiart1ev1aaatCvAUfKttLearuWrP9MDH5MBPbIqV92AaeXatLxBI9gBaebbnrfifHhDYfgasaacH8akY=wiFfYdH8Gipec8Eeeu0xXdbba9frFj0=OqFfea0dXdd9vqai=hGuQ8kuc9pgc9s8qqaq=dirpe0xb9q8qiLsFr0=vr0=vr0dc8meaabaqaciaacaGaaeqabaqabeGadaaakeaacqWGnbqtdaWgaaWcbaGaemyAaKMaemOAaOMaem4AaSgabeaakiabg2da9maaqahabaGaemOzayMaeiikaGIaemyAaKMaeiilaWIaemOAaOMaeiilaWIaem4AaSMaeiilaWIaemiBaWMaeiykaKcaleaacqWGSbaBcqGH9aqpcqaIXaqmaeaacqWGUbGBa0GaeyyeIuoakiaaxMaacaWLjaWaaeWaaeaacqaI0aanaiaawIcacaGLPaaaaaa@490C@

where *n *is the number of classes of distances. *σ *is the weight given to each observation. *σ *= 1/50 is used for smoothing [19]. fkij(l)
 MathType@MTEF@5@5@+=feaafiart1ev1aaatCvAUfKttLearuWrP9MDH5MBPbIqV92AaeXatLxBI9gBaebbnrfifHhDYfgasaacH8akY=wiFfYdH8Gipec8Eeeu0xXdbba9frFj0=OqFfea0dXdd9vqai=hGuQ8kuc9pgc9s8qqaq=dirpe0xb9q8qiLsFr0=vr0=vr0dc8meaabaqaciaacaGaaeqabaqabeGadaaakeaacqWGMbGzdaqhaaWcbaGaem4AaSgabaGaemyAaKMaemOAaOgaaOGaeiikaGIaemiBaWMaeiykaKcaaa@3562@ is the relative frequency of occurrence for the interaction center type pair *ij *at sequence separation *k *in the class of distance *l*:

fkij(l)=f(i,j,k,l)Mijk     (5)
 MathType@MTEF@5@5@+=feaafiart1ev1aaatCvAUfKttLearuWrP9MDH5MBPbIqV92AaeXatLxBI9gBaebbnrfifHhDYfgasaacH8akY=wiFfYdH8Gipec8Eeeu0xXdbba9frFj0=OqFfea0dXdd9vqai=hGuQ8kuc9pgc9s8qqaq=dirpe0xb9q8qiLsFr0=vr0=vr0dc8meaabaqaciaacaGaaeqabaqabeGadaaakeaacqWGMbGzdaqhaaWcbaGaem4AaSgabaGaemyAaKMaemOAaOgaaOGaeiikaGIaemiBaWMaeiykaKIaeyypa0ZaaSaaaeaacqWGMbGzcqGGOaakcqWGPbqAcqGGSaalcqWGQbGAcqGGSaalcqWGRbWAcqGGSaalcqWGSbaBcqGGPaqkaeaacqWGnbqtdaWgaaWcbaGaemyAaKMaemOAaOMaem4AaSgabeaaaaGccaWLjaGaaCzcamaabmaabaGaeGynaudacaGLOaGaayzkaaaaaa@4ACC@

fkxx(l)
 MathType@MTEF@5@5@+=feaafiart1ev1aaatCvAUfKttLearuWrP9MDH5MBPbIqV92AaeXatLxBI9gBaebbnrfifHhDYfgasaacH8akY=wiFfYdH8Gipec8Eeeu0xXdbba9frFj0=OqFfea0dXdd9vqai=hGuQ8kuc9pgc9s8qqaq=dirpe0xb9q8qiLsFr0=vr0=vr0dc8meaabaqaciaacaGaaeqabaqabeGadaaakeaacqWGMbGzdaqhaaWcbaGaem4AaSgabaGaemiEaGNaemiEaGhaaOGaeiikaGIaemiBaWMaeiykaKcaaa@359C@ is *the *relative frequency of occurrence for all the interaction center type pairs at sequence separation *k *in the class of distance *l*:

fkxx(l)=∑i=1r∑j=1rf(i,j,k,l)∑i=1r∑j=1r∑k=1mf(i,j,k,l)     (6)
 MathType@MTEF@5@5@+=feaafiart1ev1aaatCvAUfKttLearuWrP9MDH5MBPbIqV92AaeXatLxBI9gBaebbnrfifHhDYfgasaacH8akY=wiFfYdH8Gipec8Eeeu0xXdbba9frFj0=OqFfea0dXdd9vqai=hGuQ8kuc9pgc9s8qqaq=dirpe0xb9q8qiLsFr0=vr0=vr0dc8meaabaqaciaacaGaaeqabaqabeGadaaakeaacqWGMbGzdaqhaaWcbaGaem4AaSgabaGaemiEaGNaemiEaGhaaOGaeiikaGIaemiBaWMaeiykaKIaeyypa0ZaaSaaaeaadaaeWbqaamaaqahabaGaemOzayMaeiikaGIaemyAaKMaeiilaWIaemOAaOMaeiilaWIaem4AaSMaeiilaWIaemiBaWMaeiykaKcaleaacqWGQbGAcqGH9aqpcqaIXaqmaeaacqWGYbGCa0GaeyyeIuoaaSqaaiabdMgaPjabg2da9iabigdaXaqaaiabdkhaYbqdcqGHris5aaGcbaWaaabCaeaadaaeWbqaamaaqahabaGaemOzayMaeiikaGIaemyAaKMaeiilaWIaemOAaOMaeiilaWIaem4AaSMaeiilaWIaemiBaWMaeiykaKcaleaacqWGRbWAcqGH9aqpcqaIXaqmaeaacqWGTbqBa0GaeyyeIuoaaSqaaiabdQgaQjabg2da9iabigdaXaqaaiabdkhaYbqdcqGHris5aaWcbaGaemyAaKMaeyypa0JaeGymaedabaGaemOCaihaniabggHiLdaaaOGaaCzcaiaaxMaadaqadaqaaiabiAda2aGaayjkaiaawMcaaaaa@73C9@

in which *r *is the number of different interaction center types and *m *is the number of classes for the sequence separation. The temperature *T *is set to 300 *K*, resulting in *RT *of 0.6 kcal/mole, where *R *is the gas constant.

#### Contact potential

The contact potential is obtained by the propensity of each of the interaction types for each of the contact number. The contact potential [18] is given by:

E(i,Ni)=−RTln⁡Nobs(i,k)∑kNobs(i,k)/Ncbin     (7)
 MathType@MTEF@5@5@+=feaafiart1ev1aaatCvAUfKttLearuWrP9MDH5MBPbIqV92AaeXatLxBI9gBaebbnrfifHhDYfgasaacH8akY=wiFfYdH8Gipec8Eeeu0xXdbba9frFj0=OqFfea0dXdd9vqai=hGuQ8kuc9pgc9s8qqaq=dirpe0xb9q8qiLsFr0=vr0=vr0dc8meaabaqaciaacaGaaeqabaqabeGadaaakeaacqWGfbqrcqGGOaakcqWGPbqAcqGGSaalcqWGobGtdaWgaaWcbaGaemyAaKgabeaakiabcMcaPiabg2da9iabgkHiTiabdkfasjabdsfaujGbcYgaSjabc6gaUnaalaaabaGaemOta40aaSbaaSqaaiabd+gaVjabdkgaIjabdohaZbqabaGccqGGOaakcqWGPbqAcqGGSaalcqWGRbWAcqGGPaqkaeaadaaeqbqaaiabd6eaonaaBaaaleaacqWGVbWBcqWGIbGycqWGZbWCaeqaaaqaaiabdUgaRbqab0GaeyyeIuoakiabcIcaOiabdMgaPjabcYcaSiabdUgaRjabcMcaPiabc+caViabd6eaonaaBaaaleaacqWGJbWycqWGIbGycqWGPbqAcqWGUbGBaeqaaaaakiaaxMaacaWLjaWaaeWaaeaacqaI3aWnaiaawIcacaGLPaaaaaa@5FFA@

where *i *is the interaction types (amino acids or binary profiles), *N*_*i *_is the contact number of the interaction center *i*. *N*_*obs*_*(i, k) *is the number of observed contacts of interaction center *i *with other interaction centers at *k*'th bin and *N*_*cbin *_is the number of contact bins. A contact is defined by the Ca-Ca distance of two interaction centers within 8 Å. The number of contact bins is set to 25. In the rare occasions of more than 25 contacts, the statistics is included in the bin for 25 contacts.

#### Φ/Ψ dihedral angle

The Φ/Ψ dihedral angle potential [18] is obtained by the propensity of each of the interaction types for each dihedral class. The Φ/Ψ dihedral angle potential is given by:



where *i *is the interaction type (amino acids or binary profiles), Φ_*i*_, Ψ_*i *_are the torsion angles at interaction center *i*. The torsion potential is the logarithm of the number of observed occurrence of the interaction center type *i *at torsion angles of Φ_*i*_, Ψ_*i *_[*N*_*obs*_(*i*, Φ_*i*_, Ψ_*i*_)] normalized by the averaged occurrence. Each torsional angle is divided into 36 bins. That is, *N*_*bin *_is equal to 36.

#### Accessible surface statistical potential

The accessible surface potential is calculated as described in [16,20]. The accessible surface of an interaction center is defined as the number of interaction centers within a sphere around the center interaction center. The radius of the sphere is the distance range of the potential. From these distributions, the statistical potential is calculated as follows:

E(i,Si)=−RTln⁡Nobs(i,s)∑sNobs(i,s)/Nsbin     (9)
 MathType@MTEF@5@5@+=feaafiart1ev1aaatCvAUfKttLearuWrP9MDH5MBPbIqV92AaeXatLxBI9gBaebbnrfifHhDYfgasaacH8akY=wiFfYdH8Gipec8Eeeu0xXdbba9frFj0=OqFfea0dXdd9vqai=hGuQ8kuc9pgc9s8qqaq=dirpe0xb9q8qiLsFr0=vr0=vr0dc8meaabaqaciaacaGaaeqabaqabeGadaaakeaacqWGfbqrcqGGOaakcqWGPbqAcqGGSaalcqWGtbWudaWgaaWcbaGaemyAaKgabeaakiabcMcaPiabg2da9iabgkHiTiabdkfasjabdsfaujGbcYgaSjabc6gaUnaalaaabaGaemOta40aaSbaaSqaaiabd+gaVjabdkgaIjabdohaZbqabaGccqGGOaakcqWGPbqAcqGGSaalcqWGZbWCcqGGPaqkaeaadaaeqbqaaiabd6eaonaaBaaaleaacqWGVbWBcqWGIbGycqWGZbWCaeqaaaqaaiabdohaZbqab0GaeyyeIuoakiabcIcaOiabdMgaPjabcYcaSiabdohaZjabcMcaPiabc+caViabd6eaonaaBaaaleaacqWGZbWCcqWGIbGycqWGPbqAcqWGUbGBaeqaaaaakiaaxMaacaWLjaWaaeWaaeaacqaI5aqoaiaawIcacaGLPaaaaaa@6058@

where *i *is the interaction types (amino acids or binary profiles), *S*_*i *_is the number of the interaction center *i*. N_*obs*_*(i,s) *is the observed occurrence of other interaction center with interaction center *i *at burial class *s*. *N*_*sbin *_is the total number of burial classes.

Note that the last three potentials don't use the smoothing technique that is adopted by the distance-dependent potential, since the known structures for calculating potentials are very large. We find that the potentials without smoothing have the same discriminative power as those with smoothing (data not shown).

#### Energy and energy Z-score

For distance-dependent potentials, the energy of a protein structure model is the sum of the individual terms over all interaction type pair *i *and *j*, sequence separations *k *and distance classes *l*:

Em=∑i<j,k,lE(i,j,k,l)     (10)
 MathType@MTEF@5@5@+=feaafiart1ev1aaatCvAUfKttLearuWrP9MDH5MBPbIqV92AaeXatLxBI9gBaebbnrfifHhDYfgasaacH8akY=wiFfYdH8Gipec8Eeeu0xXdbba9frFj0=OqFfea0dXdd9vqai=hGuQ8kuc9pgc9s8qqaq=dirpe0xb9q8qiLsFr0=vr0=vr0dc8meaabaqaciaacaGaaeqabaqabeGadaaakeaacqWGfbqrdaWgaaWcbaGaemyBa0gabeaakiabg2da9maaqafabaGaemyrauKaeiikaGIaemyAaKMaeiilaWIaemOAaOMaeiilaWIaem4AaSMaeiilaWIaemiBaWMaeiykaKcaleaacqWGPbqAcqGH8aapcqWGQbGAcqGGSaalcqWGRbWAcqGGSaalcqWGSbaBaeqaniabggHiLdGccaWLjaGaaCzcamaabmaabaGaeGymaeJaeGimaadacaGLOaGaayzkaaaaaa@4A4F@

For the contact, accessible surface and Φ/Ψ dihedral angle potentials, the energy of the model is the sum of the terms for all of the residues (residue-level) or binary profiles (profile-level).

Before an energy is used to discriminate between the good and bad models, it is transformed into a Z-score of energy [20]:

Z=Em−μrσr     (11)
 MathType@MTEF@5@5@+=feaafiart1ev1aaatCvAUfKttLearuWrP9MDH5MBPbIqV92AaeXatLxBI9gBaebbnrfifHhDYfgasaacH8akY=wiFfYdH8Gipec8Eeeu0xXdbba9frFj0=OqFfea0dXdd9vqai=hGuQ8kuc9pgc9s8qqaq=dirpe0xb9q8qiLsFr0=vr0=vr0dc8meaabaqaciaacaGaaeqabaqabeGadaaakeaacqWGAbGwcqGH9aqpdaWcaaqaaiabdweafnaaBaaaleaacqWGTbqBaeqaaOGaeyOeI0ccciGae8hVd02aaSbaaSqaaiabdkhaYbqabaaakeaacqWFdpWCdaWgaaWcbaGaemOCaihabeaaaaGccaWLjaGaaCzcamaabmaabaGaeGymaeJaeGymaedacaGLOaGaayzkaaaaaa@3E06@

where *E*_*m *_is the energy of the model, *μ*_*r *_and *σ*_*r *_are the average and standard deviation of the reference energy distribution respectively. Two different reference energy distributions are widely used. The first approach involves randomization of the order of residues in the tested model (sequence space reference). The second derivation of the reference energy distribution keeps the original sequence, but changes its conformation (structure space reference). Due to the similar performance [11] and the relative simplicity, the sequence space reference is applied here. The randomization procedure is repeated 200 times, generating 200 reference models.

### Performance metrics

The fractions of the false positives (FP) and false negatives (FN) are defined as:

FP=BB+D,FN=CA+C     (12)
 MathType@MTEF@5@5@+=feaafiart1ev1aaatCvAUfKttLearuWrP9MDH5MBPbIqV92AaeXatLxBI9gBaebbnrfifHhDYfgasaacH8akY=wiFfYdH8Gipec8Eeeu0xXdbba9frFj0=OqFfea0dXdd9vqai=hGuQ8kuc9pgc9s8qqaq=dirpe0xb9q8qiLsFr0=vr0=vr0dc8meaabaqaciaacaGaaeqabaqabeGadaaakeaafaqabeqacaaabaGaemOrayKaemiuaaLaeyypa0ZaaSaaaeaacqWGcbGqaeaacqWGcbGqcqGHRaWkcqWGebaraaGaeiilaWcabaGaemOrayKaemOta4Kaeyypa0ZaaSaaaeaacqWGdbWqaeaacqWGbbqqcqGHRaWkcqWGdbWqaaaaaiaaxMaacaWLjaWaaeWaaeaacqaIXaqmcqaIYaGmaiaawIcacaGLPaaaaaa@4104@

in which A is the number of true positives (good models predicted as good), B is the number of false positives (good models predicted as bad), C is the number of false negatives (bad models predicted as good) and D is the number of true negatives (bad models predicted as bad). The fraction of the Correctly Predicted (CP) cases or the correct classification rate at the optimal value of the energy Z-score cutoff is used to assess the performance of a given statistical potential in fold assessment as follows [11]:

CP=A′+D′A′+B′+C′+D′     (13)
 MathType@MTEF@5@5@+=feaafiart1ev1aaatCvAUfKttLearuWrP9MDH5MBPbIqV92AaeXatLxBI9gBaebbnrfifHhDYfgasaacH8akY=wiFfYdH8Gipec8Eeeu0xXdbba9frFj0=OqFfea0dXdd9vqai=hGuQ8kuc9pgc9s8qqaq=dirpe0xb9q8qiLsFr0=vr0=vr0dc8meaabaqaciaacaGaaeqabaqabeGadaaakeaacqWGdbWqcqWGqbaucqGH9aqpdaWcaaqaaiqbdgeabzaafaGaey4kaSIafmiraqKbauaaaeaacuWGbbqqgaqbaiabgUcaRiqbdkeaczaafaGaey4kaSIafm4qamKbauaacqGHRaWkcuWGebargaqbaaaacaWLjaGaaCzcamaabmaabaGaeGymaeJaeG4mamdacaGLOaGaayzkaaaaaa@3ECF@

in which the prime is used to indicated the corresponding values at the energy Z-score cutoff that results in the maximal correct classification rate.

Receiver operating characteristic (ROC) curves [71] are also used to assess the statistical potentials. An ROC plot is obtained by plotting the false negatives fraction against the corresponding false positives fraction for all cutoffs on the energy Z-score. The area under the ROC curve represents the probability of incorrect classification over the whole range of cutoffs, which ranges form 0 to 0.5. If it is 0.5, the scores for the good and bad models do not differ (no discrimination power), whereas a value of 0 indicates no overlap between the two sets of models (perfect discrimination).

For decoy set evaluation, two other performance metrics are adopted [42]. One is the success rate in native discriminations, which is defined as the overall ratio of native-rank as top 1 rank. The other is the Z-score of the decoy set, defined as:

Z−score=(<Edecoy>−Enative)/<(Edecoy)2>−<Edecoy>2     (14)
 MathType@MTEF@5@5@+=feaafiart1ev1aaatCvAUfKttLearuWrP9MDH5MBPbIqV92AaeXatLxBI9gBaebbnrfifHhDYfgasaacH8akY=wiFfYdH8Gipec8Eeeu0xXdbba9frFj0=OqFfea0dXdd9vqai=hGuQ8kuc9pgc9s8qqaq=dirpe0xb9q8qiLsFr0=vr0=vr0dc8meaabaqaciaacaGaaeqabaqabeGadaaakeaacqWGAbGwcqGHsislcqWGZbWCcqWGJbWycqWGVbWBcqWGYbGCcqWGLbqzcqGH9aqpcqGGOaakcqGH8aapcqWGfbqrdaahaaWcbeqaaiabdsgaKjabdwgaLjabdogaJjabd+gaVjabdMha5baakiabg6da+iabgkHiTiabdweafnaaCaaaleqabaGaemOBa4MaemyyaeMaemiDaqNaemyAaKMaemODayNaemyzaugaaOGaeiykaKIaei4la8YaaOaaaeaacqGH8aapcqGGOaakcqWGfbqrdaahaaWcbeqaaiabdsgaKjabdwgaLjabdogaJjabd+gaVjabdMha5baakiabcMcaPmaaCaaaleqabaGaeGOmaidaaOGaeyOpa4JaeyOeI0IaeyipaWJaemyrau0aaWbaaSqabeaacqWGKbazcqWGLbqzcqWGJbWycqWGVbWBcqWG5bqEaaGccqGH+aGpdaahaaWcbeqaaiabikdaYaaaaeqaaOGaaCzcaiaaxMaadaqadaqaaiabigdaXiabisda0aGaayjkaiaawMcaaaaa@6C09@

where <> denotes the average over all decoy structures, and *E*^*native *^is the energy of the native structure. Z-score is a measure of the bias toward the native structure.

## Availability

The source code is included as an additional file [see Additional file 1], can be freely downloaded at  and is available upon request from the authors.

## Authors' contributions

QD carried out the knowledge-based potential studies, participated in coding and drafted the manuscript. LL participated in the design of the study and performed the statistical analysis. XW conceived of the study, and participated in its design and coordination. All authors read and approved the final manuscript.

## Supplementary Material

Additional File 1 Source code. Source code for the profile-level statistical potentials Click here for file

## Figures and Tables

**Table 1:** Comparative results of potentials at our structure models

Potentials	CP	Success rates	Z-scores	Potentials	CP	Success rates	Z-scores
Distance	0.86	400/431	2.86	Dihedral	0.81	256/431	1.92
Distance_profile	0.91	422/431	3.26	Dihedral_profile	0.82	270/431	2.08
Contact	0.81	221/431	1.84	Surface	0.85	309/431	2.33
Contact_profile	0.83	232/431	1.96	Surface_profile	0.90	335/431	2.78

The distance, contact, dihedral and surface refer to the four kinds of potentials at the residue level. The potentials with _profile suffix indicate the corresponding potentials at the profile level. In the success rates columns, the first number is the number of native structures ranked number one; the second number is the total number of proteins in the decoy set.

**Table 2:** Comparative fold assessment results of potentials at the Baker's set

Potentials	CP	Success rates	Z-scores	Potentials	CP	Success rates	Z-scores
Distance	0.77	25/41	2.58	Dihedral	0.75	17/41	1.41
Distance_profile	0.81	30/41	2.74	Dihedral_profile	0.77	20/41	1.58
Contact	0.74	15/41	1.36	Surface	0.74	18/41	2.47
Contact_profile	0.75	17/41	1.27	Surface_profile	0.78	22/41	2.54

See the footnote of table 1 for the name of the potentials.

**Table 3:** The results of PROSTAR decoy set evaluation

Decoy set	MISFOLD	IFU	PDBERR
Number of decoy pair	25	41	3
Distance	25	28	3
Distance_profile	25	35	3
Contact	24	25	1
Contact_profile	25	28	3
Dihedral	25	26	3
Dihedral_profile	25	29	3
Surface	25	21	1
Surface_profile	25	24	3

Given in the table are the number of decoy pair and correctly recognized decoy pair for all potentials on the three decoy sets. See the footnote of table 1 for the name of the potentials.

**Table 4:** The success rates and the average Z-scores of different potentials on the multiple decoy sets

Source	4state	Lattice_ssfit	Lmds	Fisa	Fisa_casp3	Summary
DFIRD-SCM	6/7 (3.94)^a^	8/8 (6.19)	3/10 (2.56)	3/4 (4.70)	3/3 (6.05)	23/32 (4.68)
Distance	5/7 (2.48)	6/8 (4.97)	2/10 (1.78)	2/4 (3.06)	1/3 (1.93)	16/32 (2.84)
Distance_profile	7/7 (3.53)	8/8 (5.72)	3/10 (2.45)	2/4 (3.32)	2/3 (2.94)	22/32 (3.59)
Contact	3/7 (1.38)	4/8 (2.32)	1/10 (0.83)	0/4 (0.65)	0/3 (1.69)	8/32 (1.37)
Contact_profile	3/7 (1.52)	5/8 (2.96)	1/10 (1.15)	0/4 (0.72)	0/3 (1.73)	9/32 (1.61)
Dihedral	7/7 (2.69)	6/8 (3.51)	2/10 (1.62)	1/4 (1.05)	1/3 (1.72)	17/32 (2.12)
Dihedral_profile	7/7 (2.72)	7/8 (3.88)	3/10 (1.55)	1/4 (1.22)	2/3 (2.58)	20/32 (2.39)
Surface	4/7 (1.80)	4/8 (3.15)	3/10 (1.21)	1/4 (1.28)	2/3 (2.26)	14/32 (1.94)
Surface_profile	4/7 (2.07)	4/8 (3.57)	5/10 (2.68)	2/4 (1.89)	2/3 (2.96)	17/32 (2.64)

^a^The first number is the number of native structures ranked as number one; the second number is total number of proteins in the decoy set. The numbers in parentheses are the average Z-scores. The results of DFIRD-SCM method are directly taken from Zhang et al., Protein Sci. 2004, 13: 400–411.

**Table 5:** The optimized results of probability threshold.

Probability threshold	Number of profiles	Distance_profile	Contact_profile	Dihedral_profile	Surface_profile
0.04	21355	-	0.828263	0.815943	0.899127
0.05	19868	-	0.826101	0.815291	0.900184
0.06	15935	-	0.825473	0.816991	0.900127
0.08	7444	-	0.825815	0.815693	0.898678
0.10	3145	-	0.827828	0.815039	0.900998
0.12	1442	-	0.826786	0.814627	0.899441
0.14	759	0.907069	0.82626	0.816084	0.899387
0.16	404	0.909359	0.826889	0.815488	0.899437
0.17	303	0.906705	0.82597	0.81585	0.899063
0.18	235	0.909907	0.824466	0.816644	0.899639
0.20	186	0.908468	0.828051	0.815918	0.899407
0.22	138	0.906744	0.823012	0.811962	0.896226
0.24	81	0.907125	0.825444	0.81052	0.895877
0.26	46	0.904767	0.823669	0.809967	0.896212
0.28	28	0.892552	0.816189	0.799421	0.887908
0.30	21	0.873879	0.782432	0.777818	0.867548
0.32	21	0.872684	0.781907	0.779257	0.866134

Given in the table are the average CP scores of profile-level statistical potentials at different probability threshold. The discrimination is performed on our structure models. The distance_profile, contact_profile, dihedral_profile and surface_profile refer to the profile-level statistical potentials of distance-dependent, contact, Φ/Ψ dihedral angle and accessible surface respectively. Note that for small *P*_*h *_value (<0.12), the profile-level distance-dependent potentials cannot produce efficient output, because the parameters of this potential are proportional to the square of the number of profiles.

**Table 6:** The results of two threading methods

Method	W_0_	W_1_	W_s_	Training accuracy	Test accuracy
Residue-threading	4.5	0.5	0.175	78.2%	75.6%
Profile-threading	5	0.4	0.348	82.5%	79.4%

W0, W1 and Ws are the gap-open penalty, gap-extension penalty and the structure factor. The training accuracy and test accuracy are the alignment accuracy on the training set and test set.
